# Evaluating modeling approaches for estimating net ecosystem exchange in Canadian peatlands

**DOI:** 10.1038/s41598-026-67275-6

**Published:** 2026-08-24

**Authors:** E. Kathryn Hettinga, Fereidoun Rezanezhad, Ali Reza Shahvaran, Mir Amir Mohammad Reshadi, Maria Strack, Kyle A. Arndt, Ian B. Strachan, June Skeeter, Jianghua Wu, Matthew Q. Morison, Haley Alcock, Oliver Sonnentag, Sara H. Knox, Rosemary Howard, Philippe Van Cappellen

**Affiliations:** 1 Ecohydrology Research Group, Department of Earth and Environmental Sciences, University of Waterloo, Waterloo, Canada; 2 Water Institute, University of Waterloo, Waterloo, Canada; 3 Department of Geography and Environmental Management, University of Waterloo, Waterloo, Canada; 4 Woodwell Climate Research Center, Falmouth, USA; 5 Department of Geography and Planning, Queen’s University, Kingston, Canada; 6 Geologic Survey of Canada, Natural Resources Canada, Vancouver, Canada; 7 School of Science and the Environment, Memorial University of Newfoundland, Corner Brook, Canada; 8 Department of Geography, University of Winnipeg, Winnipeg, Canada; 9 Département de Géographie, Université de Montréal, Montreal, Canada; 10 Department of Geography and Environmental Studies, Wilfrid Laurier University, Waterloo, Canada; 11 Department of Geography, University of British Columbia, Vancouver, Canada; 12 Department of Geography, McGill University, Montreal, Canada

**Keywords:** Peatlands, Net ecosystem exchange (NEE), Carbon dioxide (CO_2_), Eddy covariance (EC), Remote sensing, Machine learning, Climate sciences, Ecology, Ecology, Environmental sciences, Hydrology

## Abstract

Peatlands are globally significant carbon reservoirs, yet models for peatland carbon cycling are often limited to the site level. To enable the prediction of carbon dioxide (CO_2_) fluxes in peatlands where no in situ measurements exist, machine learning algorithms must be trained on in situ CO_2_ flux measurements from multiple sites. In this study, year-round eddy covariance (EC)-derived net ecosystem exchange (NEE) measurements and 32 hydroclimatic predictor variables were compiled for 21 Canadian peatland sites spanning seven ecoregions. A comprehensive feature selection workflow carried out on the predictor variables (features) identified that model performance stabilized at four features, which were: evapotranspiration, burn area index, normalized difference water index, and modeled soil moisture. Four machine learning algorithms: ElasticNet Regression (EN), Light Gradient-Boosting Machine (LGBM), Random Forest Regression (RF), and Support Vector Regression (SVR) were trained and evaluated using held-out test data. The best performing model was the LGBM model, which was then assessed for generalizability via a leave-one-ecoregion-out sensitivity analysis, which highlighted the necessity of ensuring predictor variables fall within the range of the training data before applying this model framework to additional sites. These findings offer a framework for regional scaling to improve Canada’s spatially explicit CO_2_ emission estimates.

**Supplementary Information:**

The online version contains supplementary material available at https://doi.org/10.1038/s41598-026-67275-6.

## Introduction

Peatlands are globally significant organic carbon (C) reservoirs, storing up to one third of the terrestrial soil organic C pool despite covering just 3% of Earth’s land surface^1–3^. This extraordinary accumulation of organic C reflects greatly reduced decomposition under the anoxic, water-saturated conditions characteristic of peat soils^4,5^. In these ecosystems, gross primary productivity (GPP), the integrated photosynthetic uptake of carbon dioxide (CO_2_) by vegetation, generally exceeds ecosystem respiration (ER), the release of CO_2_ by plants and microorganisms, yielding a net CO_2_ sink and hereto a negative net ecosystem exchange (NEE)^6^. Furthermore, in terms of peatland C budgets, CO_2_ uptake typically outweighs emissions of methane (CH_4_) and dissolved or particulate organic C^7^. Overall, peatlands are known to be small, but persistent organic C sinks, and northern peatlands in Canada typically sequester an estimated 20–60 g C m^−2^ yr^−1^^7,8^.

Two primary in situ approaches are used to measure NEE: chamber-based methods and the eddy covariance (EC) technique. In this study, data derived from the EC technique was used. This method leverages micrometeorological theory using fast-response sensors mounted above the canopy to record rapid fluctuations in vertical wind speed and CO_2_ concentration^9^. By correlating these fluctuations, EC derives ecosystem-scale flux estimates that typically span 100 s of meters to square kilometers, offering a spatially explicit flux at the landscape scale^9^. Although EC systems require more elaborate setup and quality control processing, compared to chamber-based techniques, their ability to integrate across whole ecosystems and measure quasi-continuously and non-intrusively outweighs these considerations^10,11^.

To predict peatland CO_2_ budgets where no in situ observations exist, models must be trained using available gridded predictors. A growing body of literature demonstrates the power of combining EC observations with gridded climate data and satellite-derived products to upscale CO_2_ fluxes across landscapes. Examples include deciduous broadleaf forests of Japan^12^, grasslands of the northern Great Plains^13^, forests in Europe^14^, and diverse biomes throughout the continental United States^15^, as well as more recent global applications^16,17^. In these studies, a suite of gridded climate data and remote-sensing-derived predictor variables have proven successful for predicting NEE.

At the site‐scale, data‐driven methods have proven effective at predicting NEE across a variety of ecosystems, ranging from forests and grasslands to agricultural systems^18–21^. In peatlands specifically, several modeling paradigms have been applied at individual tower sites, for example: process‐based models^7,22^, data‐driven approaches^23^, and semi-empirical formulations^24,25^. Meanwhile, in other biomes or ecosystem types, site‐level EC observations have been expanded to regional flux estimates using data-driven approaches, for example: across the United States^15^, global FLUXNET sites^16,26,27^, and a variety of other ecosystem types at variable scales^28–31^, with similar upscaling efforts predicting CH_4_ emissions from wetlands^32–34^. By contrast, despite peatlands implicit inclusion in some of these studies, few studies have attempted to apply these regional‐scale data‐driven strategies specifically to peatland NEE, either across the Northern Hemisphere^35^ or within Canada^36^. As a starting point for future application of data-driven models on a pan-Canada scale, the performance of several models should be assessed.

In this study, year-round EC-derived NEE measurements from 21 Canadian peatland sites spanning seven ecoregions were integrated with 32 hydroclimatic predictor variables or features from remote sensing and gridded climate datasets. The main objectives were to: (i) execute a comprehensive feature importance and selection workflow to narrow inputs to the most influential drivers of NEE in Canadian peatlands; (ii) train, validate, and test four machine learning algorithms encompassing a range of complexity: ElasticNet Regression (EN), Light Gradient-Boosting Machine (LGBM), Random Forest Regression (RF), and Support Vector Regression (SVR); and (iii) assess the generalizability and regional transferability of the top-performing model. This study aims to provide a generalizable framework for upscaling NEE estimates across Canada’s peatlands.

## Materials and methods

### Data collection and processing

#### Site selection

**Fig. 1:**
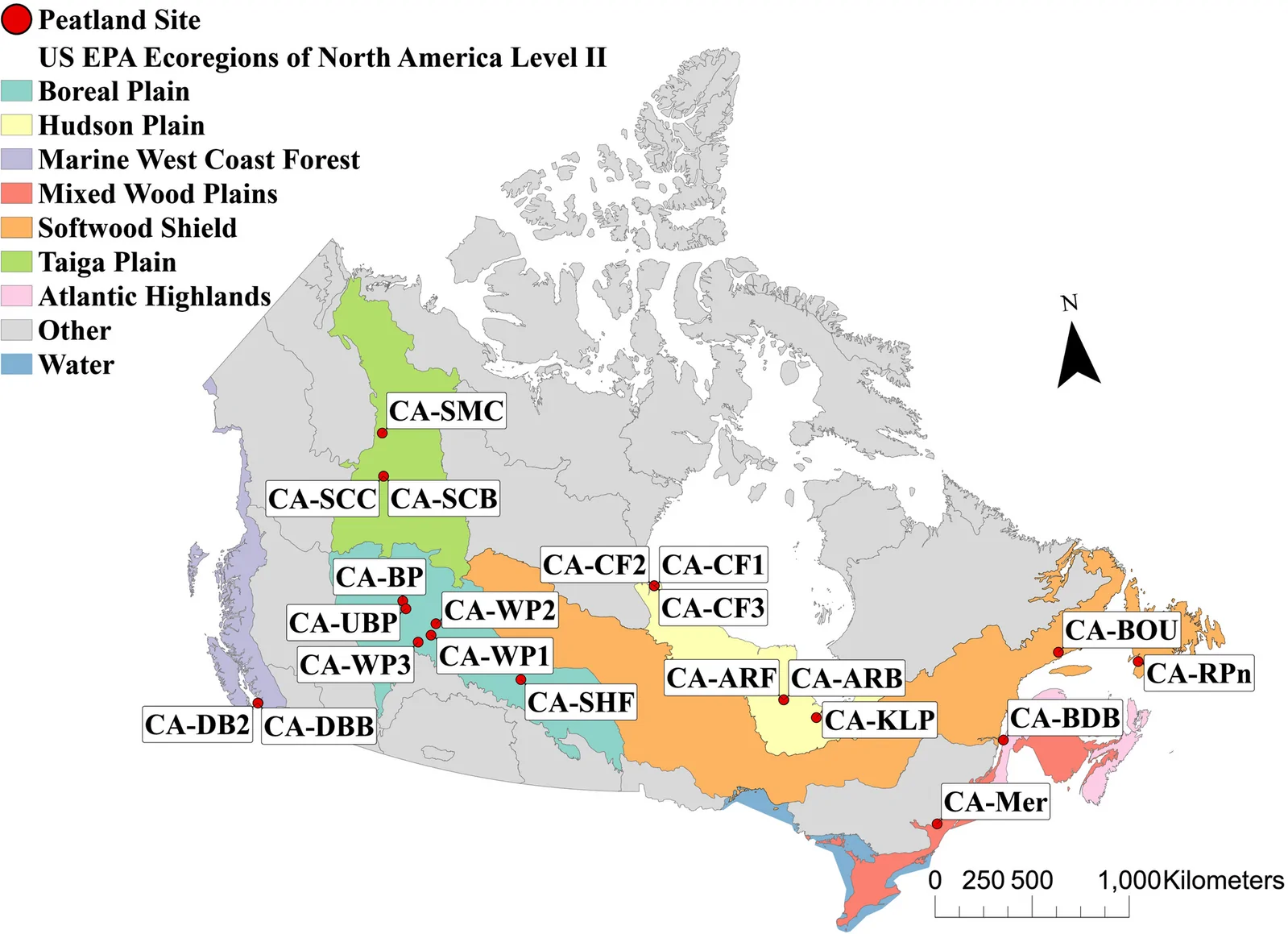
Geographic distribution of the twenty-one selected peatland sites across Canada, overlaid on the U.S. EPA Level II Ecoregions of North America^104^.

Half‐hourly NEE data of 21 Canadian peatland sites (Fig. 1 and Table 1) with continuous EC‐derived net CO_2_ flux records were obtained from the AmeriFlux network^37^, and individual site principal investigators. The data were extracted from Canadian peatland sites with available data on AmeriFlux and the Web of Science database was searched for additional peatland sites with publicly accessible or principal investigator provided NEE data. The selected sites were representative of different peatland types, including 11 bogs (ombrotrophic systems receiving water and nutrients solely from precipitation and dry deposition) and 10 fens (receiving additional water and nutrient inputs from connections with the ground and surface water)^38^. Two sites (CA-DBB and CA-DB2) within Burns Bog Ecological Conservancy Area represent historically disturbed sites, as they were subject to peat extraction from 1957 to 1963 along with additional disturbance via land use change in the surrounding urban area. These sites have undergone phased restoration since 2001 and now function as overall CO_2_ sinks^39–42^. Another site (CA-BDB) was subject to peat extraction by vacuum harvest between 1972 and 1980, but was actively restored using the moss layer transfer technique in 1999 and 2000, with the restoration returning the site to an annual CO_2_ sink comparable with a long-term reference site (CA-Mer) for the years included in this study^43,44^. Additionally, one site (CA-BP) had experienced a wildfire approximately 2–3 years before the EC-measurements included in this study but had returned to a CO_2_ sink during the period of observation^45^, while the remaining seventeen sites are generally undisturbed. Collectively, these 21 sites encompass a variety of different peatland types, and have some representation of pristine, disturbed, and restored sites, providing a robust foundation for training and evaluating the predictive models. EC system configurations for each site are detailed in Supplementary Table S1.

**Table 1:** Characteristics of the selected 21 peatland study sites: site identifier, geographic coordinates, EPA Level II ecoregion, Köppen climate classification, mean annual temperature and precipitation, peatland hydrological type, and primary data citation^37,89^.

AmeriFlux Site ID	Coordinates (°)		US EPA level II ecoregion	Canadian ecozone	Köppen climate classification*	Mean annual temperature (°C)	Mean annual precipitation(mm)	Peatland classification	References
	Latitude	Longitude							
CA-SMC	63.1534	− 123.2522	Taiga Plain	Taiga Plains	Dfc	− 2.80	388.00	Bog	^90,91^
CA-SCB	61.3089	− 121.2984	Taiga Plain	Taiga Plains	Dfc	− 2.80	388.00	Bog	^80^
CA-SCC	61.3079	−121.2992	Taiga Plain	Taiga Plains	Dfc	−2.80	388.00	Bog	^80^
CA-ARB	52.6950	− 83.9452	Hudson Plain	Hudson Plains	Dfb	− 1.30	700.00	Bog	^81^
CA-KLP	51.5902	− 81.7684	Hudson Plain	Hudson Plains	Dfb	− 0.5	703.60	Bog	^92^
CA-BOU	50.5244	− 63.2064	Softwood Shield	Boreal Shield	Cwc	1.50	1011.00	Bog	^93^
CA-DBB	49.1293	− 122.9849	Marine West Coast Forest	Pacific Maritime	Csb	10.00	1128.00	Bog	^39^
CA-DB2	49.1190	− 122.9951	Marine West Coast Forest	Pacific Maritime	Csb	10.00	1128.00	Bog	^41^
CA-RPn	48.2604	− 58.6632	Softwood Shield	Boreal Shield	Dfb	5.00	1340.40	Bog	^82^
CA-BDB	47.9672	−69.4287	Atlantic Highlands	Atlantic Maritime	Dfb	3.5	963.50	Bog	^43,44^
CA-Mer	45.4094	− 75.5186	Mixed Wood Plains	Mixedwood Plains	Dfb	6.00	943.00	Bog	^94^
CA-CF1	58.6658	− 93.8300	Hudson Plain	Hudson Plains	Dfc	− 6.50	452.00	Fen	^95,96^
CA-CF2	58.6658	− 93.8300	Hudson Plain	Hudson Plains	Dfc	− 6.50	452.00	Fen	^95,96^
CA-CF3	58.6658	− 93.8304	Hudson Plain	Hudson Plains	Dfc	− 6.50	452.00	Fen	^97^
CA-BP	56.1070	− 115.5610	Boreal Plain	Boreal Plains	Dfc	1.7	462.10	Fen	^45^
CA-UBP	55.8000	− 115.1000	Boreal Plain	Boreal Plains	Dfc	1.7	462.10	Fen	^45^
CA-WP2	55.5375	− 112.3343	Boreal Plain	Boreal Plains	Dfc	1.20	428.30	Fen	^98,99^
CA-WP1	54.9538	− 112.4670	Boreal Plain	Boreal Plains	Dfc	1.10	461.07	Fen	^100,101^
CA-WP3	54.4700	− 113.3200	Boreal Plain	Boreal Plains	Dfc	2.30	478.70	Fen	^98,99^
CA-SHF	53.8021	− 104.6180	Boreal Plain	Boreal Plains	Dfb	0.9	427.30	Fen	^102,103^
CA-ARF	52.7008	− 83.9550	Hudson Plain	Hudson Plains	Dfb	− 1.30	700.00	Fen	^81^

*Dfb = a continental climate with no dry season and warm summers; Cwc = a temperate climate with dry winters and cool summers; Dfc = a continental climate with no dry season and cool summers; Csb = a temperate climate with dry and warm summers .

#### Observation-based NEE fluxes

The half‐hourly NEE flux data of the 21 selected peatland sites were processed in REddyProcWeb^46^ to apply friction‐velocity (u*) filtering and gap‐filling. Low‐turbulence periods, when the EC method underestimates fluxes^47^, were identified and removed using seasonally calculated u* thresholds, after which gaps were filled via marginal distribution sampling following Falge et al.^48^ and Reichstein et al.^49^. Processed data were then aggregated to daily and monthly means using the following standards ≥ 70% half‐hourly data per day, and ≥ 10 days per month, making use of all monthly means that met this criteria. This workflow adheres to established protocols^46,47^ , with full methodological details provided in the Supplementary Materials.

#### Gridded predictor variables

Thirty-two predictor variables were selected based on their relevance to modeling NEE in peatlands (see Table 2). To capture the high temporal variability of peatland CO_2_ fluxes, static predictor variables or those with coarser-than‐monthly temporal resolution were excluded from collection. All predictor variables are accessible via Google Earth Engine (GEE)^50^ and Table 2 summarizes each predictor variable’s temporal cadence, spatial resolution, unit, and source. Given that EC flux tower footprints typically span 250–3000 m radii around the location of the tower depending on tower height^51^, predictor variables were extracted by taking the mean value of grid cells within a 1 km buffer around the tower coordinates. For coarser resolution datasets (> 5 km, the grid cell value containing the flux tower was used directly. Data were extracted in this way to account for dynamic flux footprints, to standardize the methods across different EC measurement heights, and to account for potential geolocation uncertainty of satellite grid cells. Data extraction followed each variable’s native cadence. Each daily mean NEE observation was then matched to the closest available predictor variable value within a ± 4‐day window, selecting the date with the smallest offset when multiple candidates existed. Additionally, processing steps (unit conversions and merging dual‐platform products, e.g., MODIS measurements from the Terra and Aqua satellites) were applied to ensure consistency across datasets before model development. Once all daily predictor variable matchups were compiled and harmonized, they were aggregated to monthly means, yielding a final dataset of monthly‐mean environmental predictor variables paired with monthly‐mean NEE as the target variable. Data were aggregated to monthly-mean to make use of the wealth of variables in the TerraClimate dataset (monthly resolution) (Abatzoglou et al*.*, 2018). Lastly, non-parametric statistical analyses were completed in R due to non-normal data distributions (Shapiro–Wilk test)^52^. Differences between peatland types were evaluated using a Wilcoxon rank-sum test, whereas differences among peatland sites and ecoregion were evaluated using Kruskal–Wallis rank-sum tests, followed by pairwise Wilcoxon rank-sum post-hoc tests with Benjamini–Hochberg correction for multiple comparisons.

**Table 2:** Summary of 32 candidate hydroclimatic predictor variables extracted from Google Earth Engine (GEE). GEE datasets were accessed via https://developers.google.com/earth-engine/datasets/catalog/ concatenated with the respective Image Collection ID.

Predictor variable	Shortform	Unit	Cadence	Spatial resolution (km)	GEE image collection ID	Time span
Fraction of photosynthetically active radiation (fPAR) absorbed by vegetation	fPAR	-	4 Days	0.5	MODIS_061_MCD15A3H	2002–2025
Leaf area index (LAI)	LAI					
Average daytime land surface temperature (LST)	DLST	°C	1 Day	1	MODIS_061_MOD21C1 MODIS_061_MYD21C1	2000–2025
Average nighttime land surface temperature (LST)	NLST				MODIS_061_MOD21C1 MODIS_061_MYD21C1	
Total evapotranspiration (ET)	ET_M	mm day^−1^	8 Days	0.5	MODIS_061_MOD16A2	2001–2024
Total evapotranspiration (ET) gap-filled*	ET_M				MODIS_061_MOD16A2GF	
Gross primary product (GPP)	GPP	g carbon m^−2^ day^−1^	8 Days	0.5	CAS_IGSNRR_PML_V2_v018	2001–2024
Normalized difference water index (NDWI)*	NDWI	-	1 Day	~ 0.5	MODIS_MCD43A4_006_NDWI	2000–2023
Normalized difference snow index (NDSI)	NDSI				MODIS_MCD43A4_006_NDSI	
Normalized difference vegetation index (NDVI)	NDVI				MODIS_MCD43A4_006_NDVI	
Enhanced vegetation index (EVI)	EVI				MODIS_MCD43A4_006_EVI	
Burn area index (BAI)*	BAI				MODIS_MCD43A4_006_BAI	
Duration of the daylight period	Dayl	hr	1 Day	1	NASA_ORNL_DAYMET_V4	1980–2024
Daily total precipitation	prcp_DM	mm				
Incident shortwave radiation flux density	Srad_DM	W m^−2^				
Snow water equivalent	swe_DM	kg m^−2^				
Daily maximum 2-m air temperature	Tmax_DM	°C				
Daily minimum 2-m air temperature	Tmin_DM	°C				
Daily average partial pressure of water vapor	vp_DM	kPa				
Actual evapotranspiration	aet	mm	Monthly	~ 5	IDAHO_EPSCOR_TERRACLIMATE	1958–2024
Climate water deficit	cwd	mm				
Palmer drought severity index (PDSI)	PDSI	-				
Potential evapotranspiration	pet	mm				
Precipitation accumulation	prcp_TC	mm				
Runoff	ro	mm				
Soil moisture*	sm	mm				
Downward surface shortwave radiation	srad_TC	W m^−2^				
Snow water equivalent	swe_TC	mm				
Maximum temperature	Tmax_TC	°C				
Minimum temperature	Tmin_TC	°C				
Vapor pressure	vp_TC	kPa				
Vapor pressure deficit	vpd	kPa				
Wind-speed at 10m	ws	m s^−1^				

*Indicates selected feature.

### Applied machine learning models

#### ElasticNet (EN) regression

All models were implemented using Scikit-Learn libraries in Python^53^. ElasticNet (EN) is a multiple linear regression model that brings together two regularization methods: the Least absolute shrinkage and selection operator (Lasso) (L1) and Ridge (L2) penalties^54^. Its strength lies in its simplicity while retaining the flexibility of more complex approaches^55^. This model has been used in a variety of studies, such as Harrison et al.^56^ for selecting predictor variables in models of CO_2_ and CH_4_ exchange from reservoir systems, and Kujawska et al.^55^ for evaluating and predicting CO_2_ flux from agricultural soils under various treatments.

#### Light gradient-boosting machine (LGBM)

Light Gradient-Boosting Machine (LGBM) is an optimized implementation of gradient-boosting decision trees (GBDT) designed for high efficiency and scalability^57^. Like conventional GBDT, it builds decision trees sequentially, with each tree correcting the errors of its predecessors to achieve high predictive accuracy^57^. However, traditional GBDT approaches can be computationally expensive, so LGBM incorporates two key techniques: gradient-based one-side sampling and exclusive feature bundling to reduce both the number of data instances and features used during training^57^.

#### Random forest (RF) regression

Random forest regression is an ensemble learning method that constructs a multitude of decision trees using bootstrap samples of the training data and random subsets of predictor variables at each split^58^. By injecting randomness in both the sampling of observations and the selection of features, individual trees become decorrelated, dramatically reducing overfitting compared to single‐tree models^58^. During prediction, RF’s output is obtained by averaging the predictions of all constituent trees, thereby stabilizing variance and improving generalization. Beyond its use in conjunction with LGBM, RF algorithms have been successfully applied to model greenhouse gas fluxes in wetland environments, including CH_4_ emissions in boreal and temperate peatlands^32,33^, as well as other C exchange studies across diverse ecosystem types^18,30^.

#### Support vector regression (SVR)

Support Vector Regression (SVR) is a powerful extension of the Support Vector Machine (SVM) framework, repurposed for regression rather than classification. In SVR, input vectors are first mapped into a high-dimensional feature space via a nonlinear function, enabling the model to capture complex, nonlinear relationships in the data. The training of an SVR model involves solving a convex optimization problem that simultaneously minimizes model complexity and penalizes deviations beyond a user-specified tolerance of error.

### Modeling workflow

#### Feature selection

Hierarchical clustering-based exploratory data analysis performed using the SciPi python package revealed substantial redundancy among predictor variables, underscoring the need for dimensionality reduction^59^. In this study, Pearson’s and Spearman’s pairwise correlations were computed and feature pairs with correlation coefficients (*ρ *) *≥ *0.85 were flagged (Supplementary Figure S1). Mutual information scores, which quantify the amount of information that a predictor variable provides about the target variable (NEE), were then computed for each feature, and within each highly correlated pair the feature with the lower mutual information score was discarded. This procedure reduced the predictor variable set from 32 to 10 variables. Next, a forward feature selection protocol, adapted from McNicol et al.^32^, was implemented to identify the optimal feature set from the 10 remaining variables. In this forward feature selection, the four models (SVR, EN, LGBM, RF) were trained, with default model hyperparameters, on all possible combinations of two features. Mean Mean Absolute Error (MAE) was computed across all models, and the pair resulting in the lowest mean MAE was selected. In subsequent steps, all individual features were evaluated and the feature that when added to the previously selected features resulted in the lowest mean MAE across all models was selected. This forward feature selection continued until there were no remaining features. Only training data were used in this feature selection step, with performance metrics generated based on a 5-fold cross-validation of the training subset, each fold serving once as a validation set while the remaining four folds were used for fitting, and the best combination was chosen by averaging performance across folds.

#### Model development and evaluation

The modeling workflow used in this study was built on Reshadi et al.^60^ with two main adaptations: EN was included alongside LGBM, RF, and SVR, and interpretation was expanded with a more comprehensive SHAP (SHapley Additive exPlanations)‐based interpretation. For each model, the data were split into a training set (75%) and a hold‐out test set (25%), stratified by site. Hyperparameters were tuned using five‐fold cross-validation on the training data. Model performance on the unseen test data was assessed using six statistical metrics (MAE, MSE = mean squared error, RMSE = root mean squared error, MAPE = median absolute percentage error, *R*^*2*^ = coefficient of determination, WI = Willmott’s d-index).

To quantify uncertainty in model predictions and guard against overfitting, the Jackknife + resampling technique was applied^61^, which iteratively leaves out one observation, fits the model on the remaining *n-1* samples, and predicts the held-out point; these *n* predictions form a distribution from which a prediction interval is constructed.

To evaluate bias in model predictions, residual analyses were conducted to quantify the differences between observed and predicted values. Residuals were plotted against their observed values to visualize model bias at different ranges of observed data, and mean residuals were calculated for each quantile of observed values. The Locally Estimated Scatterplot Smoothing (LOESS) bias trends were also calculated for the data, given the variability of model performance based on range^62^. This strategy allows for more accurate trend evaluation for non-linear data and works especially well when the relationship between variables (observed and predicted) varies across different values of the data.

#### Model interpretation

To elucidate each predictor variable’s influence on NEE estimates, Shapley additive explanation (SHAP) analysis was applied^63^, which assigns contribution values that capture both local (individual prediction) and global (overall model) feature importance. In addition, a leave-one-ecoregion-out sensitivity analysis was conducted by iteratively omitting each ecoregion from the training data, retraining the model, and evaluating performance changes to assess the model’s reliance on specific regional data.

## Results

### Distribution of net ecosystem exchange

**Fig. 2:**
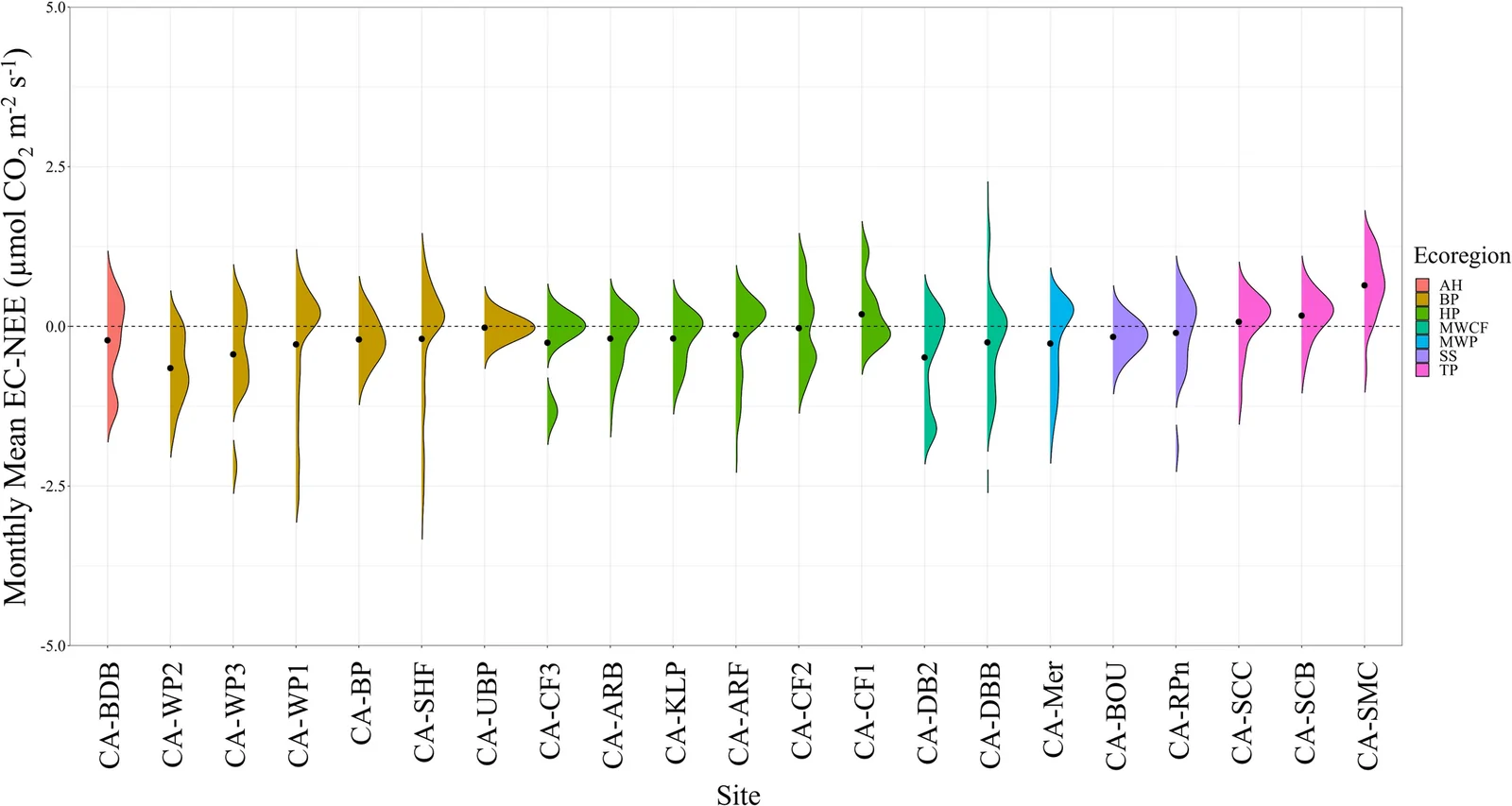
Ridgeline distributions of monthly mean eddy covariance net ecosystem exchange (EC‐NEE; µmol CO_2_ m^-2^ s^-1^) at each peatland site (*x*-axis labels). Horizontal dashed line at zero distinguishes uptake versus release. Ecoregions are defined as follows: AH = Atlantic Highlands; BP = Boreal Plain; HP = Hudson Plain; MWCF = Marine West Coast Forest; MWP = Mixed Wood Plains; SS = Softwood Shield; TP = Taiga Plain. Site names are defined in Table 1.

**Fig. 3:**
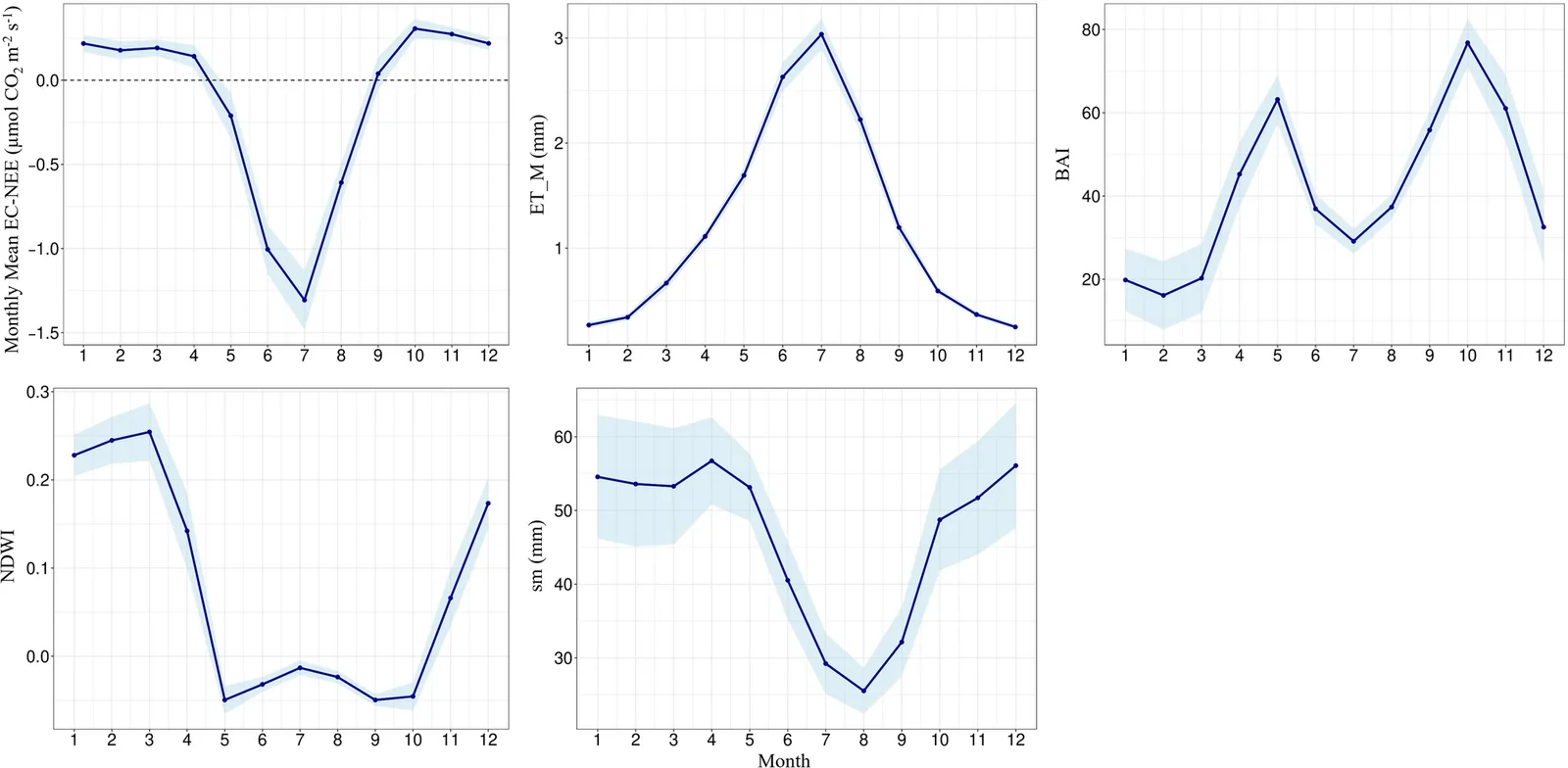
Seasonal cycles of monthly-mean net ecosystem exchange (NEE) and the four key predictor variables: evapotranspiration (ET_M), burn area index (BAI), normalized difference water index (NDWI), and modeled soil moisture (sm), with 95% confidence intervals (CI). Monthly means were computed from daily means (requiring ≥ 70% half-hourly coverage per day and ≥ 10 days per month).

The monthly mean EC-NEE fluxes ranged from -3.40 to 2.09 µmol CO_2_ m^-2^ s^-1^ (mean = -0.17 µmol CO_2_ m^-2^ s^-1^; 95% CI: − 0.22 to − 0.13; *n* = 979; median = 0.06 µmol CO_2_ m^-2^ s^-1^) (Fig. 2). Although some site-level means differed significantly (Supplementary Table S1), there were no significant differences between bogs (mean =  − 0.15 µmol CO_2_ m^-2^ s^-1^; 95% CI: − 0.20 to − 0.10; *n* = 599) and fens (mean =  − 0.21 µmol CO_2_ m^-2^ s^-1^; 95% CI: − 0.30 to − 0.13; *n* = 380) (Wilcoxon *W* = 110,768, *p* = 0.48). The Taiga Plain ecoregion showed significantly more positive monthly mean EC-NEE fluxes compared to all other ecoregions (Supplementary Table S2). NEE displayed pronounced seasonality: strong net CO_2_ uptake (negative NEE) occurred May through August, while net emission (positive NEE) prevailed during the remainder of the year (January–April and September–December) (Fig. 3).

### Distribution of predictor variables

Model performance improved sharply up to four features, after which MAE decreased indicating a decrease in model performance upon the inclusion of additional features (Supplementary Figure S2). Of the 32 candidate features initially assembled (Table 2), the feature selection workflow indicated that model performance stabilized once four features were retained: evapotranspiration (ET_M), burn area index (BAI), normalized difference water index (NDWI), and modeled soil moisture (sm) (Supplementary Figure S2). Seasonally, ET_M peaked during the summer months, while sm and NDWI reached their minimum at that time (Fig. 3). BAI exhibited an early-season peak around April–May, declined through summer, then rose again to maxima in October–November (Fig. 3). Substantial differences among all four variables were evident at both the site and ecoregion levels (Supplementary Table S2).

### Model performance

**Fig. 4:**
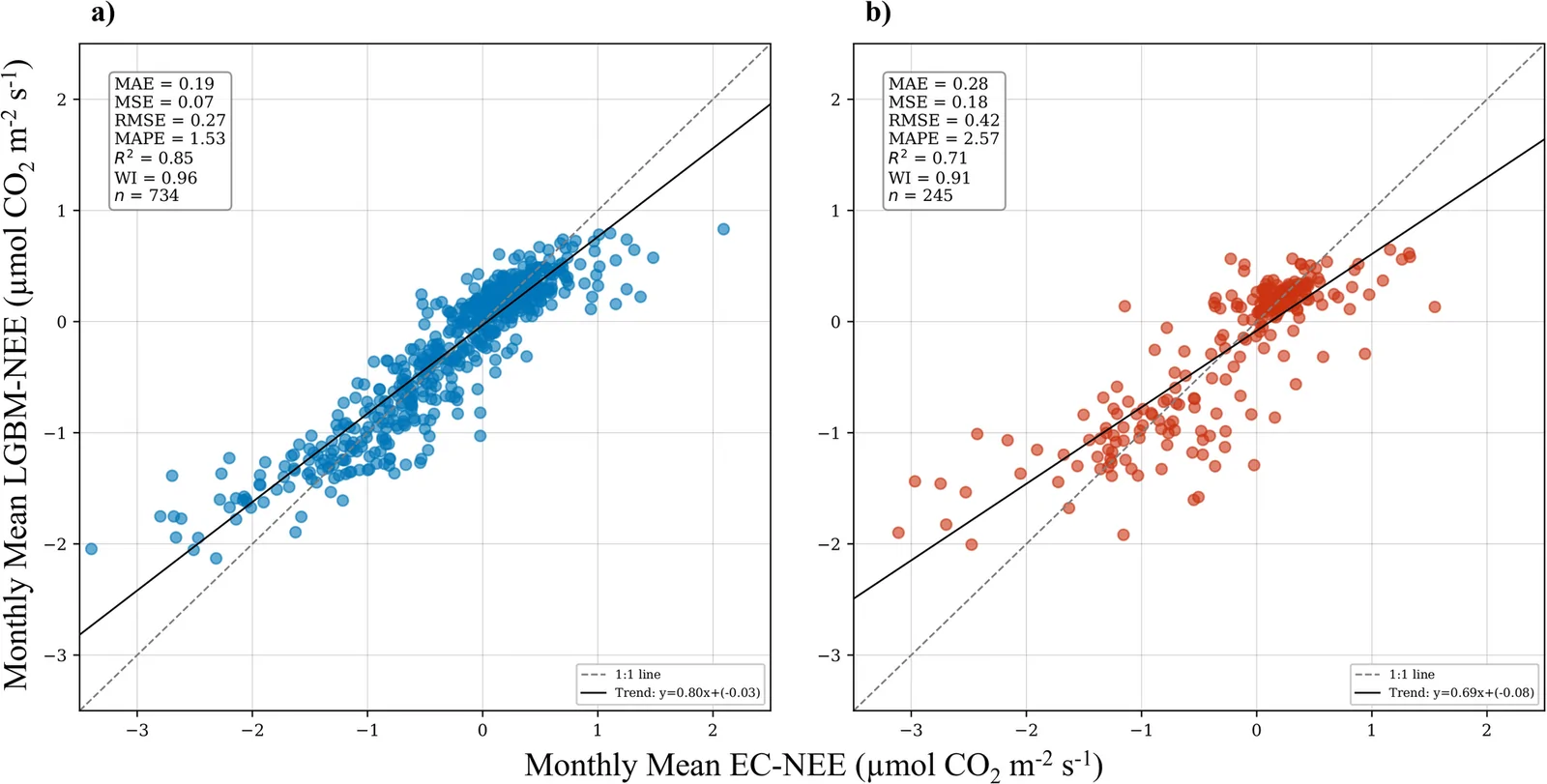
Light gradient-boosting machine (LGBM) predictions versus eddy covariance (EC) observations of daily CO_2_ net ecosystem exchange (NEE) for (**a**) the training set (*n* = 734) and (**b**) the test set (*n* = 245). Colored points show site‐level monthly means, the dashed gray line is the 1:1 reference, and the solid black line is the fitted regression. Key performance metrics (WI, *R*^*2*^, MAE, MSE, RMSE, MAPE) are listed in the inset for each panel.

**Fig. 5:**
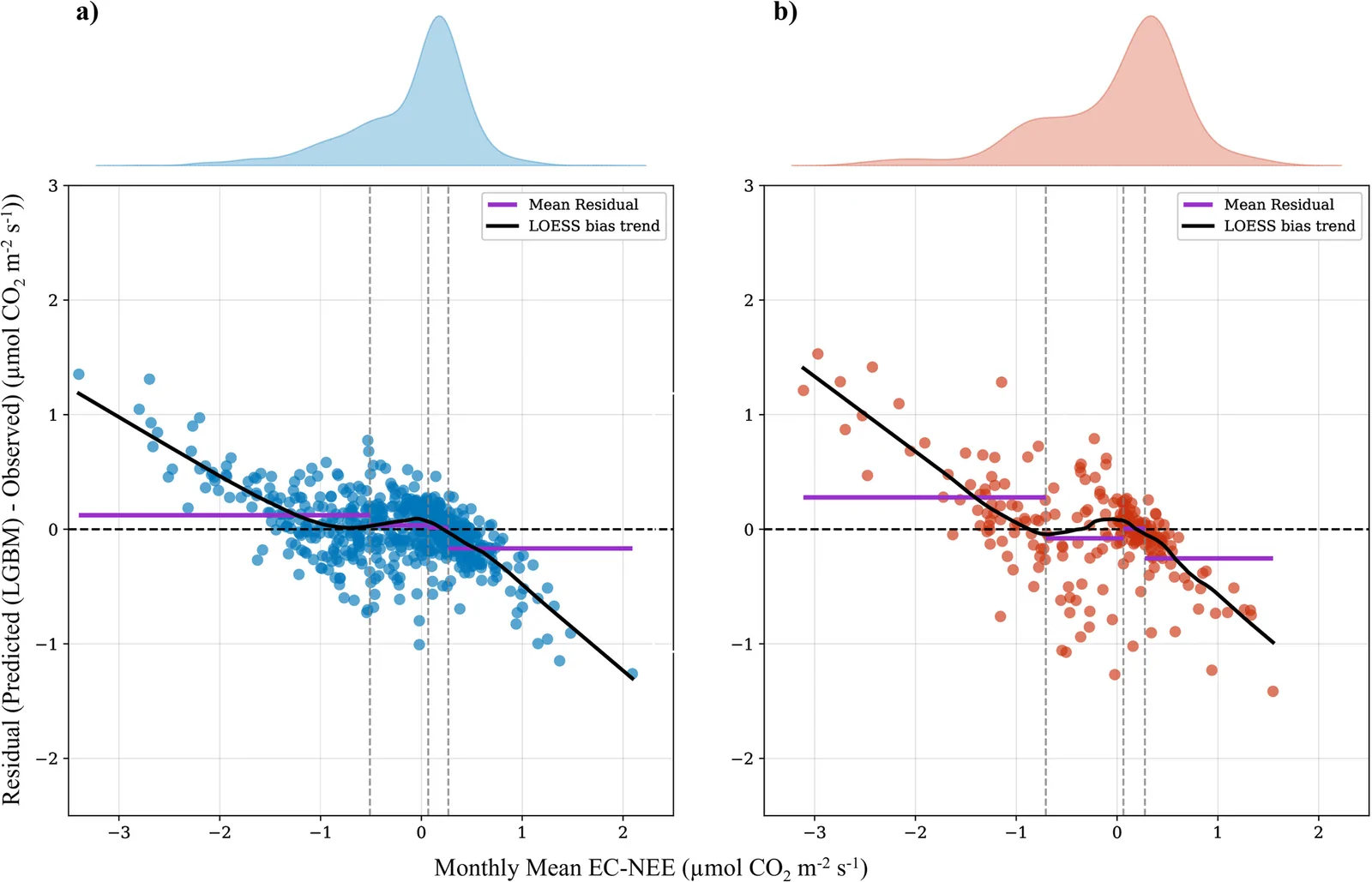
Residual (predicted – actual) plots for predictions based on the Light Gradient-Boosting Machine (LGBM) model against monthly mean EC-NEE for (**a**) the training set (*n* = 734) and (**b**) the test set (*n* = 245). Colored points show site‐level monthly means, the vertical dashed grey line marks the quantiles of data, whereas the horizontal dashed grey line marks 0. The solid black line represents the Locally Estimated Scatterplot Smoothing (LOESS) bias trend, whereas the purple solid line represents the mean residual for each quantile. The distribution of data is shown above the plots.

After training each model on these four features, performance was broadly comparable across RF, LGBM, and SVR, with EN performing worst (Fig. 4 and Supplementary Figure S3). RF and LGBM demonstrated similar performance across several of the performance metrics evaluated on the testing data (i.e., MAE, MSE, and *R*^*2*^). However, LGBM achieved marginally improved performance across the remaining performance metrics evaluated (i.e., RMSE, MAPE, and WI), which led to its selection for subsequent analyses (Table 3 and Fig. 4). All models exhibited a slight bias: they tended to predict less negative NEE when observed values were strongly negative and predict less positive NEE when observed values were strongly positive, with this bias increasing modestly from training to testing data (Fig. 5 and Supplementary Figure S4). To quantify uncertainty, prediction intervals were computed at multiple confidence levels and evaluated for coverage (the fraction of test observations whose true values fell within the interval) and mean interval width. At the 95% confidence level (*α * = 0.05), LGBM achieved a coverage of 0.94, meaning 94% of observed values lay within its intervals, with a mean interval width of 2.03 µmol CO_2_ m^-2^ s^-1^ (Supplementary Figure S5).

**Table 3:** Performance metrics on testing (and training in parentheses) data for each machine learning model. Bold values indicate the optimal performance for each metric among all models. The top-performing model, considering all metrics, is LGBM (*EN* ElasticNet regression; *LGBM* light gradient-boosting machine; *RF* random forest regression; *SVR* support vector regression).

	EN	LGBM	RF	SVR
MAE (µmol CO_2_ m^-2^ s^-1^)	0.37 (0.32)	**0.28** (0.19)	**0.28** (0.19)	0.30 (0.24)
MSE (µmol CO_2_ m^-2^ s^-1^)	0.31 (0.23)	**0.18** (0.07)	**0.18** (0.08)	0.21 (0.15)
RMSE (µmol CO_2_ m^-2^ s^-1^)	0.55 (0.48)	**0.42** (0.27)	0.43 (0.29)	0.45 (0.38)
MAPE (%)	3.96 (2.79)	**2.57** (1.53)	2.74 (1.74)	2.61 (2.12)
*R*^*2*^	0.50 (0.53)	**0.71** (0.85)	**0.71** (0.83)	0.66 (0.70)
WI	0.79 (0.82)	**0.91** (0.96)	0.90 (0.95)	0.88 (0.90)

### Model interpretation

**Fig. 6:**
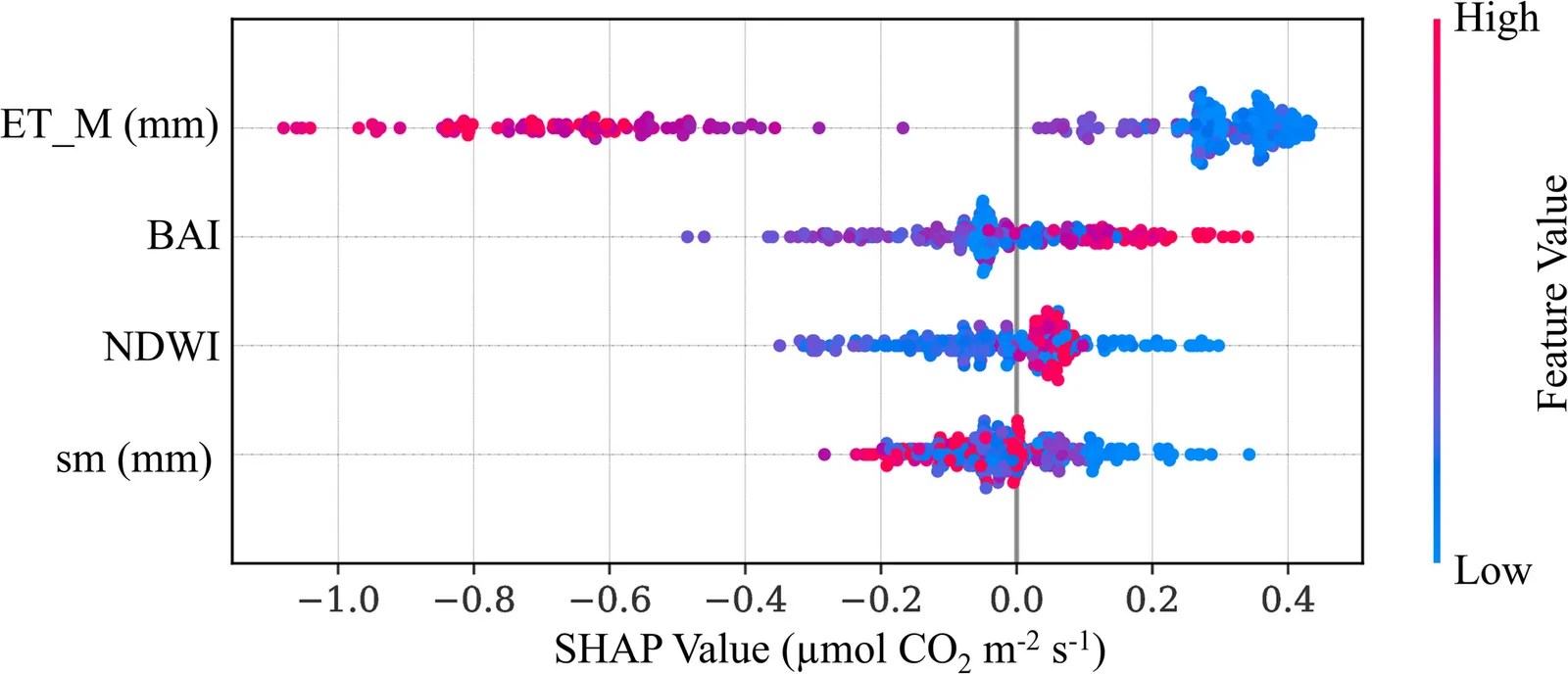
Beeswarm summary of SHAP (SHapley Additive exPlanations)-based local explanations for the selected four features: evapotranspiration (ET_M), burn area index (BAI), normalized difference water index (NDWI), and modeled soil moisture (sm), ranked by descending influence on NEE. Point colors (blue to red) show feature magnitude (low to high, respectively), the *x*-axis shows SHAP values (positive = higher predicted NEE), and each point is an individual monthly mean record illustrating feature impacts on model predictions .

SHAP analysis aids in understanding the contribution of the predictor variables on the target variable. This analysis ranks the predictor variables on their importance in driving changes in the target variable (i.e., NEE). The primary driver of NEE in the LGBM model was ET_M (SHAP value = 0.43; normalized relative contribution = 59.7%), followed by BAI (0.11; 15.2%), NDWI (0.10; 13.9%), and sm (0.08; 11.1%) (Supplementary Figure S6). The SHAP analysis also shows the effect of variables on the target (i.e., NEE) (Fig. 6). Generally, high values of ET_M and sm had a negative contribution on the prediction, while low values had a positive contribution. Conversely, low values of BAI had a negative contribution on the prediction and high values had a positive contribution. NDWI’s relationship with the prediction was more variable, with both lower and higher values having a negative contribution on the prediction, but values closer to zero having a positive contribution on the prediction. In the SHAP heatmap (Supplementary Figure S7), it is shown that all features have strongly non-linear effects on the prediction.

Partial dependence plots (Supplementary Figure S8) showed that higher ET_M corresponded to more negative NEE (i.e., greater CO_2_ uptake), whereas BAI drove predictions toward more positive NEE. NDWI and sm exhibited strongly non-linear effects: NDWI exhibited a threshold response with NEE decreasing sharply (enhanced CO_2_ uptake) up to 0.05, and sm initially enhanced uptake, but past about 20 mm net CO_2_ uptake decreased and this pattern repeated several times. SHAP interaction networks (Supplementary Figure S9) further revealed that feature interconnections were strongest in samples with the most negative NEE, suggesting these predictor variables capture growing-season dynamics more effectively.

The leave-one-ecoregion out sensitivity analysis quantified generalizability by omitting each ecoregion in turn and retraining the LGBM model (Table 4). The lowest MAE occurred when Mixed Wood Plains (MWP) (*n* monthly mean observations = 161) was excluded (MAE = 0.26 µmol CO_2_ m^-2^ s^-1^), followed by Hudson Plain (HP) ((*n* = 143; MAE = 0.27 µmol CO_2_ m^-2^ s^-1^), Atlantic Highlands (AH) (*n* = 27; MAE = 0.28 µmol CO_2_ m^-2^ s^-1^). Excluding Taiga Plain (TP), Softwood Shield (SS), Boreal Plain (BP), and Marine West Coast Forest (MWCF), all increased error above the overall mean MAE (0.35 µmol CO_2_ m^-2^ s^-1^) (Table 4). The highest error observed when the Marine West Coast Forest was omitted can be attributed to significantly different distributions of BAI, NDWI, and sm compared to the other ecoregions (Supplementary Table S2). Despite SS sharing similar distributions of BAI, ET_M, and NDWI with other ecoregions, its unique sm distribution might lead to increases in model error (Supplementary Table S2). Similarly, TP also had a unique sm distribution and significantly more positive NEE compared to other ecoregions that may skew model performance (Supplementary Table S2). The increase in model error associated with omitting BP is likely related to sample size, since distributions of predictors and NEE were similar to other ecoregions (Supplementary Table S2).

**Table 4:** Performance metrics for the leave-one-ecoregion-out sensitivity analysis.

Ecoregion	Number of observations (training, monthly)	MAE (µmol CO_2_ m^-2^ s^-1^)	MSE (µmol CO_2_ m^-2^ s^-1^)	RMSE (µmol CO_2_ m^-2^ s^-1^)	MAPE (%)	WI	*R*^*2*^
Boreal Plain	224	0.40	0.33	0.58	3.59	0.82	0.56
Hudson Plain	143	0.27	0.15	0.39	5.19	0.82	0.33
Atlantic Highlands	27	0.28	0.13	0.36	1.25	0.91	0.67
Marine West Coast Forest	61	0.46	0.35	0.59	3.64	0.84	0.39
Mixed Wood Plains	161	0.26	0.11	0.33	0.95	0.93	0.75
Softwood Shield	28	0.45	0.30	0.55	5.57	0.65	-1.34*
Taiga Plain	90	0.35	0.23	0.48	1.04	0.51	-0.07*

*Negative *R*^*2*^ values can arise in machine learning applications when a model is evaluated on held-out data, reflecting poor generalization. In this case, a negative *R*^*2*^ indicates that the model performs worse than simply predicting the mean of the target variable.

## Discussion

### Model performance and applicability

The LGBM model outperformed the other three ML approaches tested for predicting NEE at the 21 peatland sites. LGBM has also demonstrated strong predictive skill in various previous studies for peatland applications: fire-hotspot^64^, mapping vegetation^65^,and diffuse solar radiation variability^66^. These studies benchmarked LGBM against alternatives such as Random Forest (RF), Extreme Gradient Boosting, Categorical Boosting, neural networks, and k-nearest neighbour. In this study, RF also demonstrated good predictive accuracy (ranking second), narrowly behind LGBM. However, LGBM’s capacity to capture complex nonlinear relationships and correct errors made by earlier trees likely explains its superior performance relative to RF, which builds decision trees independently. Cai et al.^18^ evaluated the performance of GBM and RF for predicting NEE and highlighted that the best performance was obtained using an ensemble approach, suggesting that integrating these models into a combined ensemble could leverage the strengths of both methods, an area worth investigating in future work.

The LGBM model used in this study exhibited a modest bias: it tended to underpredict the magnitude of strong CO_2_ uptake (i.e., predicting less negative NEE than observed) and to underpredict emissions when EC‐NEE was strongly positive (Fig. 5 and Supplementary Figure S4). This is a common systematic bias for many machine learning algorithms trained on highly variable data, as they aim to minimize MSE or variance^67^. The systematic bias results in a tendency to overestimate when the observed value is small (in this case, underpredicting the magnitude when values are strongly negative), and underestimate when values are large^67^. This bias is more pronounced on highly variable data with values that deviate substantially from the mean (i.e., NEE data) but could be mitigated by correcting the systematic bias of the model with the constrained optimization method proposed by Lee and Chen^67^. Moreover, this bias increased slightly, along with a drop in performance metrics, when moving from training to testing. This drop is expected, resulting from the nature of the unseen test data and differences in sample size^68^. Additionally, machine learning models struggle to predict values outside of the range of their training data (i.e., extrapolation), where linear models have been shown to have comparable if not superior performance for extrapolation^69^. As the goal for this modeling exercise was to test different models on their efficacy in predicting peatland NEE and determine if these methods could be applied to sites where no in situ data exist (i.e., extrapolation), the EN algorithm was included with the hopes that it may be more reliable for extrapolation compared to the other three traditional machine learning algorithms tested. However, the performance metrics for both the training and testing subsets using the EN algorithm were substantially lower than the three other algorithms (Table 3 and Supplementary Figure S3). As a result, applying this workflow for upscaling might result in an underestimation of the C sink capacity of Canada’s peatlands.

The leave-one-ecoregion-out sensitivity analysis revealed that omitting individual ecoregions from the training set led to only modest increases in prediction error, demonstrating the potential for the model to be applied to ecoregions beyond the training dataset and fill regional data gaps, provided the distributions for the predictors and NEE fall within the range of the training data. However, it is crucial to highlight that there were different sample sizes represented in each ecoregion, and it is possible that sample size effects and site-specific influences may be impacting the results of the sensitivity analysis. Together, these results affirm the potential for this workflow to be applied to under- or un-represented peatland regions that share similar environmental characteristics with the original training sites.

### Predictor variables

All four final predictor variables displayed pronounced seasonality, either peaking or reaching their minimum with the seasonal cycle of peatland NEE (Fig. 3), and three of these, including ET_M, NDWI, and sm directly relate to peatland moisture status. Because the water cycle tightly couples to C cycling, the presence or absence of water can strongly limit both photosynthesis and ecosystem respiration^70–73^ and it is unsurprising that the SHAP analysis identified ET_M as the most important driver in predicting NEE. The partial dependence analysis showed that higher ET_M corresponds to more negative (stronger uptake) NEE (Supplementary Figure S8), consistent with greater latent-heat losses under well-watered conditions enhancing plant productivity (enhanced GPP)^74^. However, it is also important to note that ET_M and NEE likely have shared drivers, including leaf area index, fraction of photosynthetically active radiation, surface reflectance/albedo, land cover, solar radiation, air temperature, and vapor pressure deficit, which are all used to derive the ET_M product^75^. These shared drivers may explain the apparent importance of ET_M, as the model may be using it as a proxy that implicitly captures the effects of several correlated variables, rather than relying on each individual feature explicitly.

Next, BAI ranked second overall in the SHAP analysis, which was surprising as the BAI was designed to map recent fire scars, and none of the 21 peatland sites experienced a fire during their observation periods. BAI may have emerged as a significant feature for two possible reasons. The first being that the surface-burn signatures captured in the red and near-infrared bands for BAI are analogous to NDVI’s detection of vegetation browning^30,76,77^. It is possible that BAI, as a vegetation index, outcompeted the other vegetation indices in the feature selection process as they may have provided less unique information than BAI. Additionally, since BAI was designed to evaluate the post-burn charcoal signatures, it’s also possible that it may mislabel spaces with low reflectance, i.e., water bodies or the presence of water as burned area^78^. This finding aligns much more closely with the seasonal cycle observed for BAI, which shares similarities with a peatland’s annual hydrograph (Fig. 3). Moreover, a dendrogram shows more similarity of BAI to water indices (NDWI, NDSI, swe_DM, and swe_TC) compared to the vegetation indices (NDVI and EVI) (Supplementary Figure S10). BAI has not been included as a feature for predicting NEE in peatlands in prior data-driven modeling studies but may provide unexpected yet-helpful information about surface water presence.

While, BAI might detect the presence of water on the landscape, NDWI is related to the presence of liquid water in the vegetation canopy and is complimentary to other vegetation indices such as NDVI and EVI^79^. The seasonal cycle of NDWI closely matches that of NEE, however, NDWI likely primarily reflects seasonal variation in peatland moisture content rather than directly capturing C flux dynamics. Moreover, sm, a variable derived from a simple water-balance model, may not be representative of the sm conditions at the site. Instead, like NDWI, it’s possible that this derived sm product shared seasonal cycle with NEE led to its overall feature importance. The TerraClimate model derives sm as the balance between water availability and atmospheric demand (i.e., evapotranspiration), which leads to high values during the winter when evapotranspiration is low (Abatzoglou et al*.* 2018). This approach would not necessarily be representative of real-world winter conditions, since it does not account for the availability of this sm to plants or microorganisms (Abatzoglou et al*.* 2018). While the SHAP analysis aims to shed light on feature importance, due to the nature of black-box machine learning models it is not possible to derive mechanistic insights^63^.

### Uncertainties and limitations

Data collected via the EC method are derived from high‐frequency measurements of turbulence and CO_2_ concentrations and require extensive post‐processing^9,47–49^. Although EC provides near‐continuous half‐hourly flux estimates, instrumentation constraints and quality‐control procedures resulted in gaps amounting to 15–77% of the half-hourly data at the 21 selected sites (Supplementary Table S1). To fill these gaps, empirical gap‐filling was applied using in situ measurements including shortwave radiation, air temperature, and vapor pressure deficit, following established protocols (Marginal Distribution Sampling)^46–49^. This approach was necessary to make use of the full in situ dataset, including both NEE measurements and the meteorological data used for gap-filling at these sites. Without incorporating gap-filled data, the available training and testing data for the models would be severely limited. While using gap-filled data introduces potential drawbacks, as portions of the dataset have already been estimated through modeling, the use of these data for constructing annual budgets is the standard practice at the site level^41,45,80–82^. When the objective is to estimate fluxes in locations lacking in situ measurements (both NEE and meteorological), the use of gap-filled data is appropriate, as it reflects the estimates that would be generated at the site under standard practices. Tramontana et al.^27^ implemented identical quality‐control and gap‐filling workflows and reported lower bias when only a small fraction of data were gap‐filled. Moffat et al.^83^ similarly reported negligible bias from filling short gaps in forest ecosystems. In this study, potential bias was further minimized by confining gap‐filling to short intervals and by aggregating the resulting half‐hourly fluxes first to daily, then to monthly, means. However, limitations of eddy covariance systems (i.e., power outages associated with limited light availability and colder temperatures) result in a higher proportion of data gaps during winter months compared to summer months. Despite the applied gap-filling methodology and monthly aggregation, this seasonal bias remained in the data used in this study, with nearly twice as many observations during summer months compared to winter months across ecoregions (Supplementary Table S3). This seasonal bias was generally consistent among ecoregions, although the total number of observations varied among ecoregions. Despite this imbalance, the model did not show higher error when predicting positive NEE values (characteristic of colder months) compared to negative NEE values (characteristic of warmer months) (Figs. 4 and 5).

Furthermore, EC footprint size and position shift dynamically with turbulence and wind direction^51^. The predictor variables in this study, spanned spatial resolutions from 0.5 to 5 km, and the values were extracted within a 1 km radius around each tower location in GEE. Xiao et al.^15^ and Tramontana et al.^27^ instead averaged over a central 3 km window within 7 km MODIS scenes with the aim to capture larger footprint extents and account for potential geolocation errors. However, expanding the buffer radius introduces a trade-off: while a larger buffer can better encompass footprint heterogeneity, it also increases mixed-pixel effects that reduce spatial precision. Using a 1 km buffer for finer-resolution datasets likely incorporated some grid cells representing non-peatland areas, but this effect is minor relative to the inherent spatial mismatch and heterogeneous land-surfaces already integrated within the coarser products (e.g., TerraClimate at ~ 5 km resolution). Moreover, each data product carries its own uncertainties and constraints. Remotely sensed datasets are prone to noise, biases, and data gaps, and their retrievals can be severely impacted by clouds and snow cover^84–86^. However, sensors with high revisit frequency, such as MODIS, provide enough repeat observations to effectively gap-fill cloud-obscured intervals^87,88^. Moreover, the remotely sensed products used in this study are primarily higher-level products, which are highly processed often modeled outputs or the results from processing lower-level data, thereby minimizing noise from the sensor or surrounding pixels^87^

## Conclusion

In this study, machine learning models, trained on mean monthly EC CO_2_ fluxes from 21 Canadian peatland sites and 32 remote sensing and gridded climate predictor variables, predicted mean monthly-NEE across the country’s diverse ecoregions. Among the four algorithms tested (ElasticNet (EN), Light Gradient-Boosting Machine (LGBM), Random Forest Regression (RF), and Support Vector Regression (SVR)), LGBM emerged as the top performer using just four features: evapotranspiration, burn area index, normalized difference water index, and modeled soil moisture. By pinpointing the four most influential variables, this study shows how remote sensing and climate products can effectively complement limited in situ measurements, enabling broadscale application to Canadian peatlands. Crucially, the leave-one-ecoregion out sensitivity analysis revealed that the model maintained comparable skill when applied to different ecoregions, highlighting the potential for the model to be applied in underrepresented ecoregions provided the range of predictors are within that of the training dataset. This approach reduces reliance on costly and energy-intensive in situ measurements, and these results offer a potential pathway to predict NEE data for peatland sites across Canada where no in situ measurements exist. This workflow could deliver spatially explicit insights to inform policy and management decisions for peatland conservation and climate change mitigation across Canada.

## Supplementary Information

Below is the link to the electronic supplementary material.

Supplementary Material 1

## Data Availability

The majority of the eddy covariance data that support the findings of this study are available from AmeriFlux under the names used in the study: CA-SMC ( [Site Info for CA-SMC- AmeriFlux](https:/ameriflux.lbl.gov/sites/siteinfo/CA-SMC) ), CA-SCB ( [Site Info for CA-SCB- AmeriFlux](https:/ameriflux.lbl.gov/sites/siteinfo/CA-SCB) ), CA-SCC ( [Site Info for CA-SCC- AmeriFlux](https:/ameriflux.lbl.gov/sites/siteinfo/CA-SCC) ), CA-ARB ( [Site Info for CA-ARB- AmeriFlux](https:/ameriflux.lbl.gov/sites/siteinfo/CA-ARB) ), CA-ARF ( [Site Info for CA-ARF- AmeriFlux](https:/ameriflux.lbl.gov/sites/siteinfo/CA-ARF) ), CA-KLP ( [Site Info for CA-KLP- AmeriFlux](https:/ameriflux.lbl.gov/sites/siteinfo/CA-KLP) ), CA-BOU ( [Site Info for CA-BOU- AmeriFlux](https:/ameriflux.lbl.gov/sites/siteinfo/CA-BOU) ), CA-DBB ( [Site Info for CA-DBB- AmeriFlux](https:/ameriflux.lbl.gov/sites/siteinfo/CA-DBB) ), CA-DB2 ( [Site Info for CA-DB2- AmeriFlux](https:/ameriflux.lbl.gov/sites/siteinfo/CA-DB2) ), CA-RPn ( [Site Info for CA-RPn- AmeriFlux](https:/ameriflux.lbl.gov/sites/siteinfo/CA-RPn) ), CA-Mer ( [Site Info for CA-Mer- AmeriFlux](https:/ameriflux.lbl.gov/sites/siteinfo/CA-Mer) ), CA-CF1 ( [Site Info for CA-CF1- AmeriFlux](https:/ameriflux.lbl.gov/sites/siteinfo/CA-CF1) ), CA-CF2 ( [Site Info for CA-CF2- AmeriFlux](https:/ameriflux.lbl.gov/sites/siteinfo/CA-CF2) ), CA-CF3 ( [Site Info for CA-CF3- AmeriFlux](https:/ameriflux.lbl.gov/sites/siteinfo/CA-CF3) ), CA-WP1 ( [Site Info for CA-WP1- AmeriFlux](https:/ameriflux.lbl.gov/sites/siteinfo/CA-WP1) ), CA-WP2 ( [Site Info for CA-WP2- AmeriFlux](https:/ameriflux.lbl.gov/sites/siteinfo/CA-WP2) ), CA-WP3 ( [Site Info for CA-WP3- AmeriFlux](https:/ameriflux.lbl.gov/sites/siteinfo/CA-WP3) ). Additional data for CA-BDB is available from [Simulating ecosystem carbon dioxide fluxes and their associated influencing factors for a restored peatland](https:/zenodo.org/records/14455815) , whereas CA-SHF is available from [Long-term meteorological and carbon, water and energy flux data from the Boreal Ecosystem Research and Monitoring Sites, Saskatchewan, Canada | FRDR-DFDR](https:/www.frdr-dfdr.ca/repo/dataset/02fe32a8-9365-4f36-ab26-d812db164bfe) . Data for CA-BP and CA-UBP were available from Morison et al. (2020). The code is available from the corresponding author upon request.
