# Supplementary material for: Evaluating modeling approaches for estimating net ecosystem exchange in Canadian peatlands
Source: Sci Rep. 2026 Aug 24;16:33444. doi: 10.1038/s41598-026-67275-6 (PMC13690571; doi:10.1038/s41598-026-67275-6)
Supplement: Supplementary file 1 [file 41598_2026_67275_MOESM1_ESM.docx]

**Supplementary Materials**

**Evaluating modeling approaches for estimating net ecosystem exchange in Canadian peatlands**

E. Kathryn Hettinga^1,*^, Fereidoun Rezanezhad^1,2^, Ali Reza Shahvaran^1^, Mir Amir Mohammad Reshadi^1^, Maria Strack^2,3^, Kyle A. Arndt^4^, Ian B. Strachan^5^, June Skeeter^6^, Jianghua Wu^7^, Matthew Q. Morison^8^, Haley Alcock^9^, Oliver Sonnentag^9,10^, Sara H. Knox^11,12^, Rosemary Howard^12^, and Philippe Van Cappellen^1,2^

^1^Ecohydrology Research Group, Department of Earth and Environmental Sciences, University of Waterloo, Waterloo, Canada

^2^Water Institute, University of Waterloo, Waterloo, Canada

^3^Department of Geography and Environmental Management, University of Waterloo, Waterloo, Canada

^4^Woodwell Climate Research Center, Falmouth MA, United States

^5^Department of Geography and Planning, Queen’s University, Kingston, Canada

^6^Geologic Survey of Canada, Natural Resources Canada, Vancouver, Canada

^7^School of Science and the Environment, Memorial University of Newfoundland, Corner Brook, Canada

^8^Department of Geography, University of Winnipeg, Winnipeg, Canada

^9^Département de géographie, Université de Montréal, Montréal, Canada

^10^Department of Geography and Environmental Studies, Wilfrid Laurier University, Waterloo, Canada

^11^Department of Geography, University of British Columbia, Vancouver, Canada

^12^Department of Geography, McGill University, Montréal, Canada

^*^Corresponding author: E. Kathryn Hettinga

[k2hettin@uwaterloo.ca](mailto:k2hettin@uwaterloo.ca)

**Observation-Based NEE Fluxes (Further Details)**

Half-hourly net ecosystem exchange (NEE) data for each peatland site were downloaded from the AmeriFlux BASE repository (AmeriFlux, 2025) and imported into the REddyProcWeb online platform (Wutzler *et al.*, 2018) to perform three key steps: friction-velocity (*u**) filtering, gap‐filling, and partitioning of NEE into gross primary productivity (GPP) and ecosystem respiration (ER). In the eddy covariance (EC) approach, fast‐response instruments record turbulent fluctuations in vertical wind speed and CO_2_ concentration at 10–20 Hz, which are block‐averaged to half‐hourly fluxes under the assumption of fully developed turbulence (Papale *et al.*, 2006). However, this assumption breaks down during low‐turbulence conditions, most prevalent at night, leading to underestimation of NEE. To correct for this bias, REddyProcWeb implements a *u**‐filtering algorithm: the year is divided into four overlapping three‐month windows (Mar–May, Jun–Aug, Sep–Nov, and Dec–Feb, with the December window extending into the following calendar year to capture continuous winter conditions) (Wutzler *et al.*, 2018). Within each window, observations are first binned into six temperature quantiles and then each quantile’s data are subdivided into twenty *u** classes (Papale *et al.*, 2006). Any *u** class containing fewer than three nighttime observations is excluded to ensure statistical robustness (Wutzler *et al.*, 2018). For each temperature quantile, the *u** threshold is defined as the lowest *u** class whose mean nocturnal NEE reaches >99% of the mean flux observed in all higher‐*u** classes (Reichstein *et al.*, 2005; Papale *et al.*, 2006). To prevent spurious thresholds, an auxiliary check identifies if two consecutive *u** classes exhibit mean flux ≥0.95 × the average of the next ten higher‐*u** classes; if so, the lower of the two classes is selected as the threshold (Wutzler *et al.*, 2018). Candidate thresholds are then accepted only if the correlation coefficient between *u** and air temperature is weak (|*r*| < 0.4), as recommended by Papale *et al.* (2006); if no suitable threshold emerges, the 90^th^ percentile of the *u** distribution is applied. The final site‐ and season‐specific *u** threshold is taken as the median of the six quantile‐based values (Papale *et al.*, 2006).

After removing all fluxes measured below the *u** threshold, gaps in the resulting half-hourly NEE time series are filled using a three‐condition hierarchical lookup‐table method adapted from Falge *et al.* (2001) and Reichstein *et al.* (2005). Condition 1 applies when only NEE is missing but global shortwave radiation (Rg), air temperature (Tair), and vapor pressure deficit (VPD) are available, in which case missing fluxes are estimated from the average of similar meteorological conditions within a 14‐day window. Under Condition 2, when NEE and either Tair or VPD are missing but Rg is available, the same lookup approach is used within a 7‐day window. Under Condition 3, when all meteorological inputs are missing, flux gaps are linearly interpolated from adjacent hours. If a gap cannot be filled under one condition, the window is expanded until the gap is filled or until a 60-day limit is reached, beyond which gaps remain unfilled (this procedure does not fill pre-existing gaps that are 60 days or longer) (Wutzler *et al.*, 2018).

Following gap‐filling, REddyProcWeb partitions NEE into GPP and ER using its standard algorithms and then computes daily means when ≥70% of half‐hourly observations are present, and monthly means when ≥10 daily values exist. To quantify uncertainty in *u** thresholds, 200 seasonal pseudoreplicate datasets are generated per three‐month window, and thresholds are re‐estimated for each; the 5^th^, 50^th^, and 95^th^ percentiles of these estimates define the uncertainty range (Wutzler *et al.*, 2018). This end-to-end processing ensures that our half-hourly EC fluxes are rigorously quality-controlled, gap-filled, and partitioned in accordance with best practices.

**Table S1:** Eddy covariance setup and data characteristics. Different superscript letters indicate statistical significance between 15 selected sites (Kruskall-Wallis, *p*<0.05) (NEE = Net Ecosystem Exchange).

| **Site** | **Data Source** | **Instrumentation** | **Path Type** | **Height**  **(m)** | **Period** | **u* Threshold**  **(m s^−1^)** | **Half-Hourly Gap**  **(%)** | **Annual Monthly Mean NEE**  **(µmol CO_2_ m^-2^ s^-1^; 95% CI)** | **Number of Monthly Observations** | **Reference** |
| --- | --- | --- | --- | --- | --- | --- | --- | --- | --- | --- |
| CA-SMC | Ameriflux and Site PI | IRGA: LI-7200 | Closed-path | 14.9 | 2017–2022 | 0.17 | 66% | 0.64^b^  (0.43, 0.85) | 24 | Qu *et al.*, (2023) |
| CA-SCB | Ameriflux and Site PI | IRGA: EC150 | Open-path | 1.9 | 2015–2022 | 0.04 | 51% | 0.17^d^  (0.05, 0.29) | 29 | Helbig *et al.*, (2017) |
| CA-SCC | Ameriflux and Site PI | IRGA: EC150; IRGA: LI-7500 | Open-path; Open-path | 15.2; 15 | 2013–2022 | 0.13 | 43% | 0.07^d^  (-0.03, 0.17) | 67 | Helbig *et al.*, (2017) |
| CA-ARB | Ameriflux | IRGA: LI-7200 | Closed-path | 7.5 | 2011–2015 | 0.28 | 33% | -0.19^ac^  (-0.30, -0.08) | 57 | Helbig *et al.*, 2019; Humphreys *et al.* (2014) |
| CA-KLP | Site PI | IRGA: LI-7200 | Closed-path | 3 | 2015–2019 | -* | -* | -0.19^c^  (-0.29, -0.09) | 54 | Humphreys *et al.*, 2014 |
| CA-BOU | Ameriflux | IRGA: LI-7200 | Closed-path | 2.2 | 2018–2020 | 0.07 | 47% | -0.16^ace^  (-0.29, -0.04) | 14 | Primeau & Garneau, 2021; Taillardat *et al.*, 2022 |
| CA-DBB | Ameriflux | IRGA: LI-7200 | Closed-path | 1.8 | 2014–2020 | 0.05 | 37% | -0.25^ace^  (-0.44, -0.06) | 69 | Christen *et al*., (2016); Lee *et al*., (2017); D’Acunha *et al*., (2019) |
| CA-DB2 | Ameriflux | IRGA: LI-7200 | Closed-path | 2.5 | 2019–2020 | 0.05 | 15% | -0.49^ace^  (-0.97, 0.001) | 12 | Christen *et al*., (2016); Lee *et al*., (2017); D’Acunha *et al*., (2019) |
| CA-RPn | Site PI | IRGA: LI-7200 | Closed-path | 3.21 | 2014–2016 | -* | -* | -0.10^ad^  (-0.37, 0.17) | 22 | Wang *et al.* (2018) |
| CA-BDB | Site PI | IRGA: LI-7500 | Open-path | 1.3 | 2013–2016 | 0.07 | 34% | -0.22^acde^  (-0.45, 0.01) | 36 | He *et al.*, 2025; Nugent *et al.*, 2018 |
| CA-Mer | Ameriflux | IRGA: LI-7000 | Closed-path | 3.0 | 1998–2020 | 0.09 | 37% | -0.27^a^  (-0.36, -0.18) | 215 | Humphreys *et al.* (2014) |
| CA-CF1 | Ameriflux | IRGA: LI-7500 | Open-path | 3.9 | 2007–2008 | 0.08 | 72% | 0.19^abcd^  (-0.26, 0.64) | 7 | Hanis *et al.*, 2013; Hanis *et al.*, 2015 |
| CA-CF2 | Ameriflux | IRGA: LI-7500 | Open-path | 4.3 | 2008–2011 | 0.20 | 77% | -0.03^acde^  (-0.36, 0.31) | 15 | Hanis *et al.*, 2015 |
| CA-CF3 | Ameriflux | IRGA: LI-7500; IRGA: LI-7200 | Open-path; Closed-path | 2.5 | 2022–2023 | 0.07 | 45% | -0.26^acde^  (-1.01, 0.50) | 5 | Arndt & Natali, 2025 |
| CA-BP | Site PI | IRGA: LI-7500 | Open-path | 6 | 2013–2014 | -* | -* | -0.21^acde^  (-0.46, 0.05) | 9 | Morison *et al.*, 2020 |
| CA-UBP | Site PI | IRGA: LI-7500 | Open-path | 9 | 2013–2014 | -* | -* | -0.02^acde^  (-0.03, -0.01) | 7 | Morison *et al.*, 2020 |
| CA-WP2 | Ameriflux | IRGA: LI-7500 | Open-path | 3.0 | 2004–2006 | 0.05 | 48% | -0.65^e^  (-0.94, -0.37) | 15 | Glenn *et al.*, 2006; Adkinson *et al.*, 2011 |
| CA-WP1 | Ameriflux | IRGA: LI-7000 | Closed-path | 9.0 | 2003–2009 | 0.16 | 43% | -0.28^acd^  (-0.51, -0.06) | 72 | Syed *et al.*, 2006; Flanagan & Syed, 2011 |
| CA-WP3 | Ameriflux | IRGA: LI-7500 | Open-path | 3.0 | 2004–2006 | 0.15 | 56% | -0.44^ac^  (-0.86, -0.02) | 14 | Glenn *et al.*, 2006; Adkinson *et al.*, 2011 |
| CA-SHF | Site PI | IRGA: LI-7500; IRGA: LI-7200 | Open-path; Closed-path | 2.5 | 2003–2023 | 0.14 | 37% | -0.19^ad^  (-0.34, -0.05) | 182 | Helgason *et al.*, 2024; Barr *et al.*, 2025 |
| CA-ARF | Ameriflux | IRGA: LI-7200 | Closed-path | 7.5 | 2011–2015 | 0.19 | 30% | -0.13^ad^  (-0.30, 0.04) | 54 | Helbig *et al.*, 2019 |

*Datasets were processed (quality filtered and gap-filled) by site principal investigators.

**Table S2.** Monthly means (± 95% CI) of predictor variables and net ecosystem exchange (NEE), stratified by US EPA Level II ecoregion. Values bearing different superscript letters within each column differ significantly (*p* < 0.05, Kruskal-Wallis). Predictor variables are defined as follows: evapotranspiration (ET_M), burn area index (BAI), normalized difference water index (NDWI), and soil moisture (sm).

| **US EPA Level II Ecoregion** | **Number of Observations**  **(Training and Testing, Monthly)** | **NEE**  **(µmol CO_2_ m^-2^ s^-1^)** | **ET_M**  **(mm)** | **BAI** | **NDWI** | **sm**  **(mm)** |
| --- | --- | --- | --- | --- | --- | --- |
| Boreal Plain | 299 | -0.25^b^  (-0.35, -0.14) | 1.23^b^  (1.13, 1.33) | 52.6^a^  (48.9, 56.4) | 0.03^d^  (0.01, 0.04) | 36.4^de^  (33.6, 39.2) |
| Hudson Plain | 192 | -0.15^b^  (-0.22, -0.08) | 1.12^ab^  (1.00, 1.24) | 35.0^b^  (30.9, 39.1) | 0.09^a^  (0.07, 0.11) | 27.8^d^  (26.4, 29.3) |
| Atlantic Highlands | 36 | -0.22^b^  (-0.44, 0.01) | 1.71^c^  (1.31, 2.11) | 27.7^bd^  (21.5, 33.8) | 0.07^ab^  (0.03, 0.11) | 40.2^a^  (35.1, 45.4) |
| Marine West Coast Forest | 81 | -0.28^b^  (-0.46, -0.11) | 1.46^c^  (1.28, 1.65) | 75.9^c^  (68.6, 83.3) | -0.05^c^  (-0.06, -0.04) | 80.8^c^  (72.1, 89.5) |
| Mixed Wood Plains | 215 | -0.27^b^  (-0.36, -0.18) | 1.63^c^  (1.45, 1.81) | 28.2^bd^  (24.9, 31.4) | 0.06^a^  (0.04, 0.09) | 71.6^f^  (68.7, 74.5) |
| Softwood Shield | 36 | -0.13^b^  (-0.29, 0.04) | 1.87^c^  (1.53, 2.20) | 21.5^d^  (18.0, 25.0) | 0.05^ad^  (-0.0005, 0.10) | 34.1^e^  (30.2, 37.9) |
| Taiga Plain | 120 | 0.21^a^  (0.12, 0.29) | 0.88^a^  (0.78, 0.98) | 47.9^a^  (41.7, 54.0) | 0.11^b^  (0.08, 0.14) | 22.6^b^  (19.7, 25.4) |

**Table S3.** Number of monthly-aggregated observations in each month.

|  | **Total Number of Observations**  **(Training and Testing, Monthly)** | **Number of Observations in the Atlantic Highlands (AH) Ecoregion**  **(Training and Testing, Monthly)** | **Number of Observations in the Boreal Plain (BP) Ecoregion**  **(Training and Testing, Monthly)** | **Number of Observations in the Hudson Plain (HP) Ecoregion**  **(Training and Testing, Monthly)** | **Number of Observations in the Marine West Coast Forest (MWCF) Ecoregion**  **(Training and Testing, Monthly)** | **Number of Observations in the Mixed Wood Plains (MWP) Ecoregion**  **(Training and Testing, Monthly)** | **Number of Observations in the Softwood Shield (SS) Ecoregion**  **(Training and Testing, Monthly)** | **Number of Observations in the Taiga Plain (TP) Ecoregion**  **(Training and Testing, Monthly)** |
| --- | --- | --- | --- | --- | --- | --- | --- | --- |
| January | 62 | 2 | 17 | 13 | 6 | 18 | 1 | 5 |
| February | 62 | 2 | 17 | 12 | 6 | 18 | 0 | 7 |
| March | 69 | 2 | 16 | 12 | 7 | 18 | 3 | 11 |
| April | 85 | 3 | 24 | 15 | 7 | 18 | 3 | 15 |
| May | 88 | 3 | 29 | 15 | 7 | 18 | 4 | 12 |
| June | 82 | 3 | 30 | 17 | 7 | 18 | 5 | 2 |
| July | 88 | 4 | 32 | 21 | 7 | 18 | 5 | 1 |
| August | 113 | 4 | 34 | 22 | 8 | 18 | 5 | 22 |
| September | 110 | 4 | 35 | 20 | 8 | 18 | 5 | 20 |
| October | 89 | 4 | 27 | 15 | 7 | 18 | 2 | 16 |
| November | 74 | 3 | 22 | 16 | 6 | 17 | 1 | 9 |
| December | 57 | 2 | 16 | 14 | 5 | 18 | 2 | 0 |

**Figure S1:** Pairwise correlations among monthly‐mean predictor variables and net ecosystem exchange (NEE). The upper triangle displays Pearson’s $r$, the lower triangle Spearman’s $\rho$. Cell colors range from blue (−1 for strong negative) to red (+1 for strong positive). Asterisks indicate significance levels: * $p$ < 0.05, ** $p$ < 0.01, *** $p$ < 0.001. Variable shortforms are expressed in Table 2.

**Figure S2:** Evolution of mean model cost function (mean absolute error (MAE) (µmol CO_2_ m^-2^ s^-1^)) across all four models during forward feature selection. The final four selected features were evapotranspiration (ET_M), burn area index (BAI), normalized difference water index (NDWI), and soil moisture (sm), beyond which model performance decreased.

**Figure S3:** Random Forest Regression (RF, top row), Support Vector Regression (SVR, middle row), and ElasticNet Regression (EN, bottom row) predictions versus eddy covariance observations of daily CO_2_ net ecosystem exchange (NEE) for (a) the training set (*n* = 734) and (b) the test set (*n* = 245). Colored points show site‐level monthly means; the gray dashed line is the 1:1 reference; and the solid black line is the fitted regression. Key performance metrics (WI, *R^2^*, MAE, MSE, RMSE, MAPE) are listed in each inset.

**Figure S4:** Random Forest Regression (RF, top row), Support Vector Regression (SVR, middle row), and ElasticNet Regression (EN, bottom row) residual (predicted – actual) plots against monthly mean EC-NEE for (a) the training set (*n* = 734) and (b) the test set (*n* = 245). Colored points show site‐level monthly means, the vertical dashed grey line marks the quantiles of data, whereas the horizontal dashed grey line marks 0. The solid black line represents the Locally Estimated Scatterplot Smoothing (LOESS) bias trend, whereas the purple solid line represents the mean residual for each quantile. The distribution of data is shown above the plots.

**Figure S5:** Light Gradient Boosted Machine (LGBM) predictive performance for daily CO_2_ net ecosystem exchange (NEE). Observed EC-NEE (blue) and LGBM predictions (green) are plotted across validation instances, with the 95% prediction interval shown in orange (mean width = 1.91 µmol CO_2_ m^-2^ s^-1^). Pink stars mark monthly means falling outside the interval.

**Figure S6.** Bar chart of relative SHAP (SHapley Additive exPlanations) feature importance for four predictors: evapotranspiration (ET_M), burn area index (BAI), normalized difference water index (NDWI), and soil moisture (sm).

**Figure S7:** SHAP (SHapley Additive exPlanations) summary heatmap displaying the impact of four predictors: evapotranspiration (ET_M), burn area index (BAI), normalized difference water index (NDWI), soil moisture (sm), on predicted eddy covariance net ecosystem exchange (EC-NEE) of CO_2_ ($f(x)$). Observations (bottom axis) are ordered by decreasing predicted EC-NEE; cell colors denote SHAP values (red = positive, blue = negative), the right‐hand bar chart ranks features by mean absolute SHAP importance, and the top line plot traces $f(x)$ for each instance relative to its mean.

**Figure S8:** Partial dependence plots from the Light Gradient Boosted Machine (LGBM) model illustrating how each of the four key predictors: evapotranspiration (ET_M), burn area index (BAI), normalized difference water index (NDWI), and soil moisture (sm), influences daily CO_2_ net ecosystem exchange, with tick marks indicating the distribution of observed values.

**Figure S9:** SHAP (SHapley Additive exPlanations) interaction networks from the Light Gradient Boosted Machine model for (a) the most positive and (b) the most negative predicted net ecosystem exchange (NEE) samples (Note: The most positive NEE sample refers to the NEE observation that had the highest positive value, whereas the most negative NEE sample refers to the NEE observation that had the lowest negative value). Each feature, evapotranspiration (ET_M), burn area index (BAI), normalized difference water index (NDWI), soil moisture (sm), is shown as a node, its size proportional to its SHAP contribution to NEE, and edges connect features with line width scaled by their SHAP interaction strength (blue=negative, red=positive).

**Figure S10:** Dendrogram obtained from hierarchical clustering using the SciPi python package (Virtanen *et al.*, 2020). Each continuous feature (*n* = 32) is shown as initially dissimilar from each other feature. Moving upwards on the plot, similar groups are combined, with lower merge points indicating more similar features or groups.

**References**

AmeriFlux. (2025). *AmeriFlux*. <https://ameriflux.lbl.gov/>

Falge, E., Baldocchi, D., Olson, R., Anthoni, P., Aubinet, M., Bernhofer, C., Burba, G., Ceulemans, R., Clement, R., Dolman, H., Granier, A., Gross, P., Grünwald, T., Hollinger, D., Jensen, N.-O., Katul, G., Keronen, P., Kowalski, A., Lai, C. T., Law, B.E., Meyers, T., Moncrieff, J., Moors, E., Munger, J.W., Pilegaard, K., Rannik, U., Rebmann, C., Suyker, A., Tenhunen, J., Tu. K., Verma, S., Vesala, T., Wilson, K., & Wofsy, S. (2001). Gap filling strategies for defensible annual sums of net ecosystem exchange. *Agricultural and Forest Meteorology*, *107*(1), 43–69. <https://doi.org/10.1016/S0168-1923(00)00225-2>

Papale, D., Reichstein, M., Aubinet, M., Canfora, E., Bernhofer, C., Kutsch, W., Longdoz, B., Rambal, S., Valentini, R., Vesala, T., & Yakir, D. (2006). Towards a standardized processing of Net Ecosystem Exchange measured with eddy covariance technique: algorithms and uncertainty estimation. *Biogeosciences*, *3*(4), 571–583. <https://doi.org/10.5194/bg-3-571-2006>

Reichstein, M., Falge, E., Baldocchi, D., Papale, D., Aubinet, M., Berbigier, P., ... & Valentini, R. (2005). On the separation of net ecosystem exchange into assimilation and ecosystem respiration: review and improved algorithm. Global change biology, 11(9), 1424-1439. <https://doi.org/10.1111/j.1365-2486.2005.001002.x>

Wutzler T, Lucas-Moffat A, Migliavacca M, Knauer J, Sickel K, Sigut, Menzer O & Reichstein M (2018) Basic and extensible post-processing of eddy covariance flux data with REddyProc. Biogeosciences, Copernicus, 15, doi: [10.5194/bg-15-5015-2018](https://doi.org/10.5194/bg-15-5015-2018)
